# Mitral valve perforation after left lateral accessory pathway ablation: a case report

**DOI:** 10.1186/s13019-021-01710-9

**Published:** 2022-03-07

**Authors:** Mariem Jabeur, Adrien Carabelli, Peggy Jacon, Sandrine Venier, Jean-François Obadia, Pascal Defaye

**Affiliations:** 1 Arrhythmias Unit, Department of Cardiology, Grenoble University Hospital, CS 10217, 38043 Grenoble Cedex 09, France; 2 Department of Cardiovascular Surgery, Louis Pradel Hospital, 59 Boulevard Pinel, 69500 Bron, France

**Keywords:** Left lateral accessory pathway, Catheter ablation, Mitral valve perforation, Mitral valve repair, Minithoracotomy

## Abstract

**Background:**

Radiofrequency catheter ablation is considered to be a relatively safe procedure. This is an unusual case report in which severe mitral regurgitation was occurred after left lateral accessory pathway radiofrequency catheter ablation.

**Case presentation:**

A 15-year-old man without structural heart disease was referred for ablation of a left lateral accessory pathway. He was a rugby player who had lived with Wolff–Parkinson–White syndrome since 2017. In 2017, two failed extensive radiofrequency catheter ablations of a left lateral accessory pathway had been performed in another center. In June 2018, he underwent a third radiofrequency catheter ablation of a left lateral accessory pathway using an anterograde transseptal approach with an early recurrence one month later. A successful fourth procedure was performed in August 2018 using a retrograde aortic approach. Three months later, the patient presented to the hospital with atypical chest pain and dyspnea on exertion. Transthoracic echocardiography revealed severe mitral regurgitation caused by a perforation of the posterior leaflet. Given the symptoms and the severity of the mitral valve regurgitation, the decision was taken to proceed with surgical intervention. Posterior mitral leaflet perforation was confirmed intraoperatively. The patient underwent video-assisted mitral valve repair via Minithoracotomy approach.

**Conclusion:**

This case demonstrates a very rare complication of Wolff–Parkinson–White radiofrequency ablation.

## Background

Radiofrequency catheter ablation is an effective treatment of accessory pathway. It is considered to be a relatively safe procedure. This is an unusual case report in which severe mitral regurgitation was occurred after left lateral accessory pathway ablation.

## Case presentation

In August 2018, a 15-year-old athletic male with symptomatic Wolff–Parkinson–White syndrome underwent a fourth electrophysiological study and subsequent ablation for orthodromic reentrant tachycardia using a left lateral accessory pathway.

Two failed extensive radiofrequency catheter ablations of a left lateral accessory pathway had been performed in 2017 in another center. A three-dimensional mapping system was already used during the second procedure.

In April 2018, a third catheter ablation procedure using an anterograde transseptal approach was performed in our center with an early recurrence one month later.

**Fig. 1:**
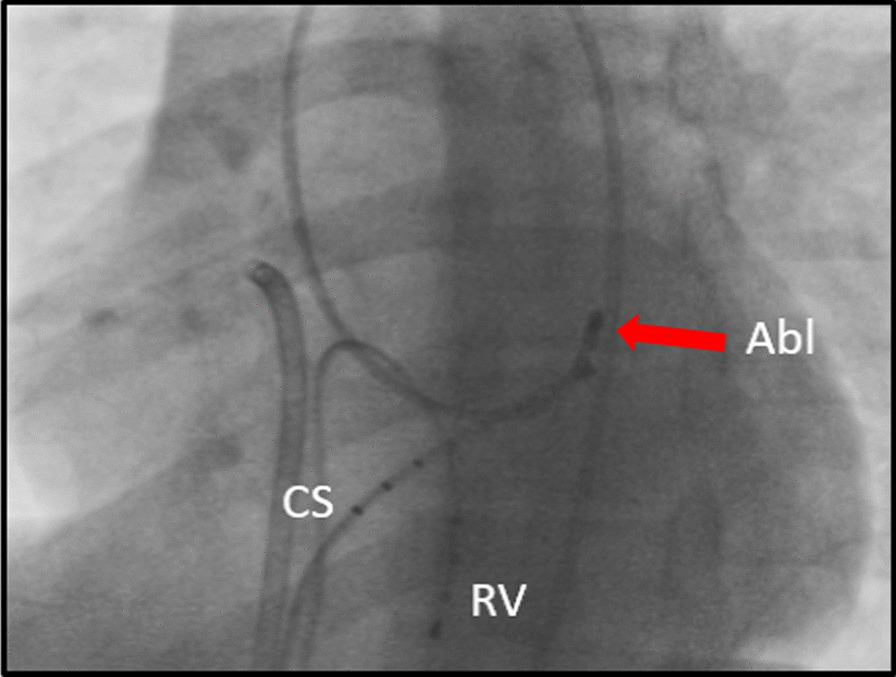
Catheter ablation position (left anterior oblique view)

A fourth ablation procedure was performed with 7French medium-curl Biosense catheter using the retrograde aortic approach (Fig. 1). Thirty-two radiofrequency lesions were delivered at the mitral valve annulus (50 W, 65 °C).

**Fig. 2:**
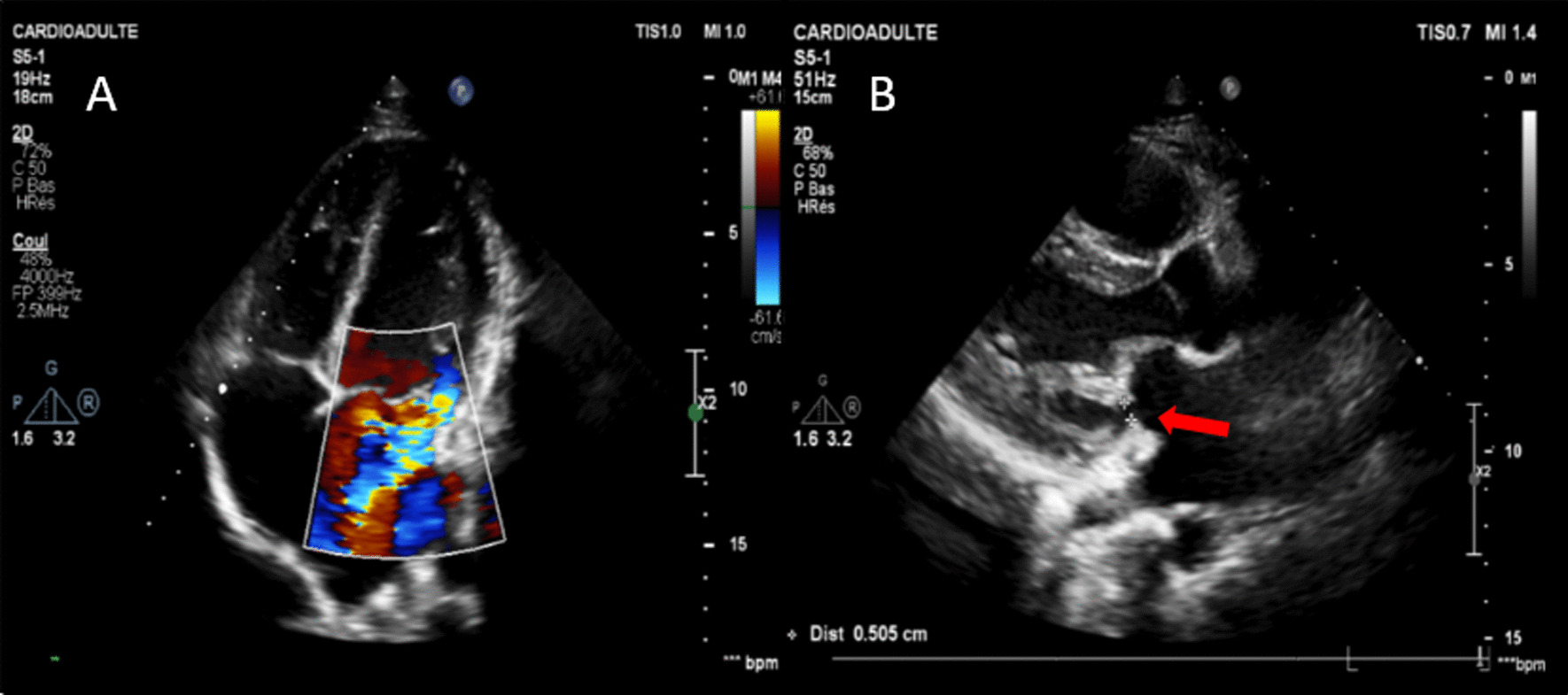
Transthoracic echocardiogram demonstrating mitral valve regurgitation; **a** four cavities view showing the eccentric jet of mitral regurgitation; **b** parasternal long-axis view showing the perforation of mitral posterior leaflet

Three months later, the patient presented to the hospital with atypical chest pain and dyspnea on exertion. Transthoracic echocardiography revealed severe mitral regurgitation with a regurgitant volume of 65 ml and an estimated regurgitant orifice of 42 mm^2^ (Fig. 2a) caused by a perforation of the posterior leaflet. This perforation measured 5 mm (Fig. 2b). The left ventricular ejection fraction was 60%.

**Fig. 3:**
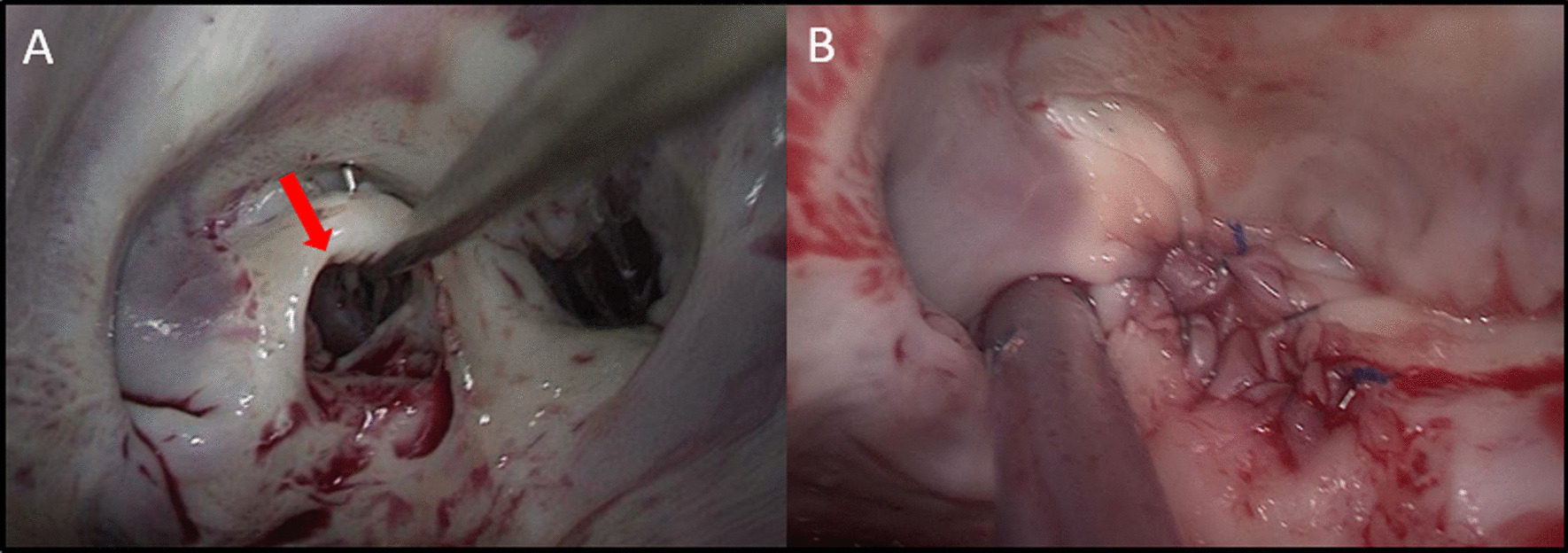
Mitral valve intraoperative photograph. **a** A perforation of the posterior leaflet; **b** Mitral valve repair

Given the symptoms and the severity of the mitral valve regurgitation, the decision was taken to proceed with surgical intervention through Minithoracotomy approach. Upon opening of the left atrium and exposure of the mitral valve, a perforation of the posterior leaflet corresponding to the medial (Fig. 3a) was confirmed. The quality of the tissue was good. The patient underwent video-assisted mitral valve repair with simple suture (Fig. 3b).

He was discharged from the hospital on postoperative day three. Postoperative transthoracic echocardiography showed mild mitral regurgitation and excellent biventricular function.

## Discussion

Radiofrequency catheter ablation is an effective treatment for Wolff–Parkinson–White syndrome in children and adults. The incidence of major complications is between 3 and 4% [1]. Compared to adults, children are at a higher risk for valvular, myocardial, and pericardial damage [2]. Ablation of the left-sided accessory pathway has the potential for injury to the mitral valve when a retrograde approach is used because the catheter is placed under the posterior leaflet of the mitral valve [1].

In one pediatric study, the incidence of mitral regurgitation after left side accessory pathway radiofrequency ablation was 9% [3]. However, the incidence of severe mitral regurgitation caused by a perforation is unknown. There are only a few cases described in the literature.

The tissue lesions could have been caused by direct thermal injury or by catheter manipulation [1].The tissue lesions caused by the radiofrequency energy may directly injure the valvar structure. The lesions are placed at the base of the leaflets, and may cause valvar insufficiency if a leaflet or a chord is trapped underneath the tip of the catheter [3].

The perforation, in this case, was likely due to direct current injury from radiofrequency. All the more so, the patient had undergone prolonged procedures with a high number of energy applications, which may have contributed to the injury. The treatment for severe mitral regurgitation is surgical reparation. Medium-term Echocardiographic follow-up is not routinely practiced after radiofrequency ablation. However, it may be useful in detecting complications following high-risk procedures that involve a large number or long duration of ablations [2].

## Conclusion

Tis case demonstrates the management of a very rare complication of Wolff–Parkinson–White radiofrequency ablation.

## Data Availability

Not applicable.
